# Deciphering podophyllotoxin-mediated defense against downy blight in *Litchi chinensis* Sonn. via integrated metabolomics and transcriptomics

**DOI:** 10.1186/s43897-026-00257-4

**Published:** 2026-09-02

**Authors:** Hailun Liu, Fachao Shi, Yingjie Wen, Yonghua Jiang, Qian Yan, Hua Huang

**Affiliations:** Institute of Fruit Tree Research, Guangdong Academy of Agricultural Sciences; Key Laboratory of South Subtropical Fruit Biology and Genetic Resource Utilization, Ministry of Agriculture and Rural Affairs; Guangdong Provincial Key Laboratory of Tropical and Subtropical Fruit Tree Research, Guangzhou, 510640 China

**Keywords:** Lychee, Lychee downy blight (LDB), Podophyllotoxin (PTOX), O-methyltransferase (OMT), Germplasm screening

## Abstract

Lychee (*Litchi chinensis* Sonn.) is a commercially important subtropical fruit. Lychee downy blight (LDB), caused by *Peronophythora litchii*, poses a major threat to lychee production. Here, we identified the LDB-resistant cultivar Yurong (YR) and the susceptible cultivar Guiwei (GW) from 288 global lychee germplasms. Widely targeted metabolomics analysis revealed a striking accumulation of podophyllotoxin (PTOX) in YR. Both in vitro and in vivo assays demonstrated that PTOX effectively inhibited the growth of *P. litchii*. Further investigation identified *LcOMT1* as a key gene encoding an O-methyltransferase (OMT) in the phenylpropanoid pathway. In resistant cultivars, a 773-bp insertion mutation (Hap3 haplotype) in the *LcOMT1* promoter enhanced its transcriptional activity. Transgenic approaches using hairy roots in lychee showed that the overexpression of either *LcOMT1* or the Hap3 haplotype led to increased PTOX biosynthesis and enhanced resistance to *P. litchii*. These results demonstrate that the Hap3-mediated upregulation of *LcOMT1* drives PTOX accumulation, establishing a crucial defense mechanism against LDB. This study provides new insights into the genetic and metabolic regulatory network underlying lychee resistance to LDB. This study may also lay a theoretical foundation for the development of PTOX-based biopesticides and offer valuable genetic resources for breeding LDB-resistant lychee cultivars.

**Supplementary Information:**

The online version contains supplementary material available at https://doi.org/10.1186/s43897-026-00257-4.

## Core

We identified a lychee cultivar (YR) resistant to LDB that accumulates high levels of PTOX, a metabolite that strongly suppresses *P. litchii* proliferation. A 773-bp insertion mutation (Hap3 haplotype) in the *LcOMT1* promoter upregulates gene transcription, which in turn promotes PTOX biosynthesis and confers enhanced LDB resistance. Our results uncover a genetic–metabolic circuit underlying LDB resistance in lychee and highlight PTOX as a promising candidate for the development of novel biocontrol agents.

## Gene and accession numbers

The sequence data were deposited in the NCBI Gene Expression Omnibus database under accession number GSE201243. The metabolomics raw data have been deposited in the OMIX database (China National Center for Bioinformation) under accession number OMIX016396.

## Introduction

Lychee (*Litchi chinensis* Sonn.) is a popular horticultural fruit planted in subtropical to tropical regions worldwide. Lychee fruit has a delicious flavor and a rich nutritional profile, including components such as carbohydrates, vitamins, and minerals, and it is a resource for the food processing and fermentation industries (Asiri et al. 2023; Zhao et al., 2020). Lychee fruit develops attractive red‒pink colors but are sensitive to water loss, pericarp browning and infection by pathogens, especially lychee downy blight (LDB) after harvest (Huang et al. 2026; Zhang et al. 2021). LDB, which is largely caused by *Peronophythora litchii*, is one of the major diseases in both lychee fruit and leaf tissues and is harmful to other subtropical to tropical plants, resulting in considerable economic losses (Gao et al. 2022). *P. litchii* growth can also hinder the development of young leaves, shoots, and branches, causing a decrease in the number of flowers and fruits (Xu et al. 2018). Consequently, the occurrence of LDB severely impacts lychee development, ripening, fruit quality and postharvest shelf life, thus greatly limiting its economic value.

Numerous reports have attempted to identify efficient methods to control LDB infection. The predominant strategy is the application of fungicides for LDB control (Tang et al., 2011). However, their high application costs, coupled with significant risks to food safety and ecological balance, limit their long-term sustainability (Wang et al. 2010). While some lychee germplasms exhibit natural resistance to LDB, systematic breeding programs aimed at developing commercial LDB-resistant cultivars have yet to be established (Sun et al. 2019). Consequently, large-scale screening of lychee germplasm to identify LDB-resistant genotypes represents an urgent priority for sustainable LDB management in lychee.

Plants have evolved diverse strategies to combat biotic stresses from pathogens (Jian et al. 2024). Beyond constitutive morphological barriers, physiological defenses integrate both preformed and inducible components orchestrated through molecular cascades (Odjakova and Hadjiivanova 2001). They involve a series of molecular events and biochemical reactions to promote the accumulation of secondary metabolites, thereby countering pathogen invasion (Zaynab et al. 2018). These mechanisms drive biochemical reprogramming to accumulate specialized metabolites, establishing chemical countermeasures against pathogen invasion (Kliebenstein 2012). Notably, despite decades of research on LDB, neither resistance-conferring metabolites nor their genetic determinants have been characterized in lychee.

Recent studies have focused on podophyllotoxin (PTOX), a lignan compound, owing to its unique chemical structure and remarkable biological activities, including antitumor and antimicrobial properties (Ardalani et al. 2017). O-Methyltransferase (OMT) serves as the cardinal regulatory node in PTOX biosynthesis, governing metabolite yield and bioactivity through stereoselective methylation of phenolic moieties on biosynthetic intermediates (Lau and Sattely 2015; Ragamustari et al. 2013). Critically, unmethylated precursors demonstrate attenuated biological potency, whereas OMT-catalyzed methylation engenders structural stabilization, which is essential for target engagement and pharmacological efficacy (Xia et al. 2000). Concurrently, functional genomic studies have revealed that the maize OMT gene confers quantitative resistance to multiple pathogens by modulating lignin biosynthesis (Yang et al. 2017). Despite these mechanistic advances, the molecular and biochemical regulation underlying LDB defense responses in lychee remains elusive.

In this study, we identified the highly LDB-resistant cultivar YR and highly susceptible cultivar GW through population-scale screening of 288 lychee landraces collected worldwide. Multiomics analyses identified the potential natural substance PTOX and key LDB resistance gene *LcOMT1* in lychee in response to *P. litchii* infection. We found that large-fragment insertion mutations in the *LcOMT1* promoter increase its transcriptional activity, thereby promoting downstream PTOX biosynthesis and conferring natural LDB resistance in lychee. This study integrates screening of LDB-resistant lychee germplasms, functional characterization of disease resistance genes, and identification of key antimicrobial metabolites. These findings will help establish a scientific foundation for breeding novel LDB-resistant cultivars and provide a mechanistic framework for the control of LDB.

## Results

### Screening of LDB-resistant varieties via population-scale antimicrobial analysis of lychee plants

**Fig. 1:**
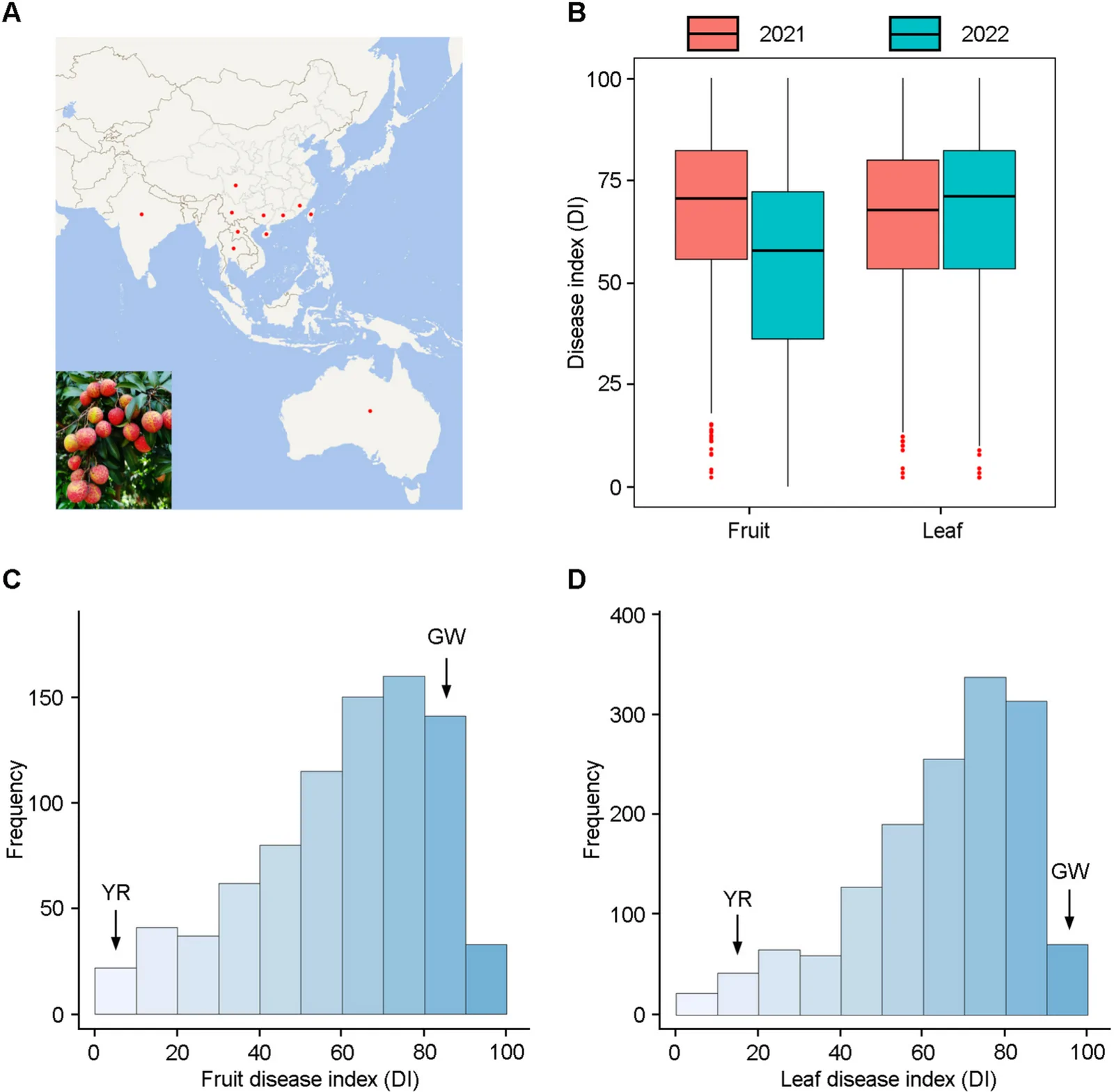
Phenotypic analysis of the DI in the natural lychee population. **A** The collection regions of the lychee germplasms (marked in red) used for this study. **B** Box plot showing the distribution of the DI of lychee based on continuous tests over two years. The bold line indicates the median value, and the shaded area represents the lower and upper quartiles. Abnormal values are depicted by red dots. **C**-**D** Frequency distributions of the DI for 288 lychee accessions in (**C**) fruits and (**D**) leaves. The arrows indicate the distributions of the LDB-resistant variety Yurong (YR) and the LDB-susceptible variety Guiwei (GW)

As an economically important fruit tree, lychee is susceptible to infection by pathogens in both its leaf and fruit tissues, with LDB posing a particular threat (Sun et al. 2019). LDB infection, an occurrence of disease in lychee, not only influences fruit quality after harvest (Zhang et al. 2021), but also negatively impacts the growth of young leaves, flowers, branches, and young fruits (Supplementary Fig. S1). To investigate the protective effects of internal substances against LDB in lychee, we screened LDB-resistant varieties from the National Lychee Germplasm Repository, where lychee germplasms are collected worldwide from southern Asia, Australia, South Africa, and other regions planted with lychee. A total of 288 lychee germplasms exhibiting various phenotypes for LDB resistance were selected from different geographic areas (Fig. 1A, Supplementary Table S1). The descriptive statistics of the DIs related to phenotypic changes were relatively stable over two consecutive years of the test, indicating an internal intrinsic response to LDB in the fruit and leaf tissues of lychee (Fig. 1B, Supplementary Table S3). However, the degree of disease infection varied significantly among the tested germplasms, indicating wide genetic variation in the population (Figs. 1C, D). The LDB-resistant variety Yurong (YR, our new cultivated variety) and the LDB-sensitive variety Guiwei (GW, a widely cultivated variety in South China) were selected as the comparative materials for further investigations (Figs. 1C, D).

### Revalidation of the susceptibility of the selected lychee varieties to LDB

**Fig. 2:**
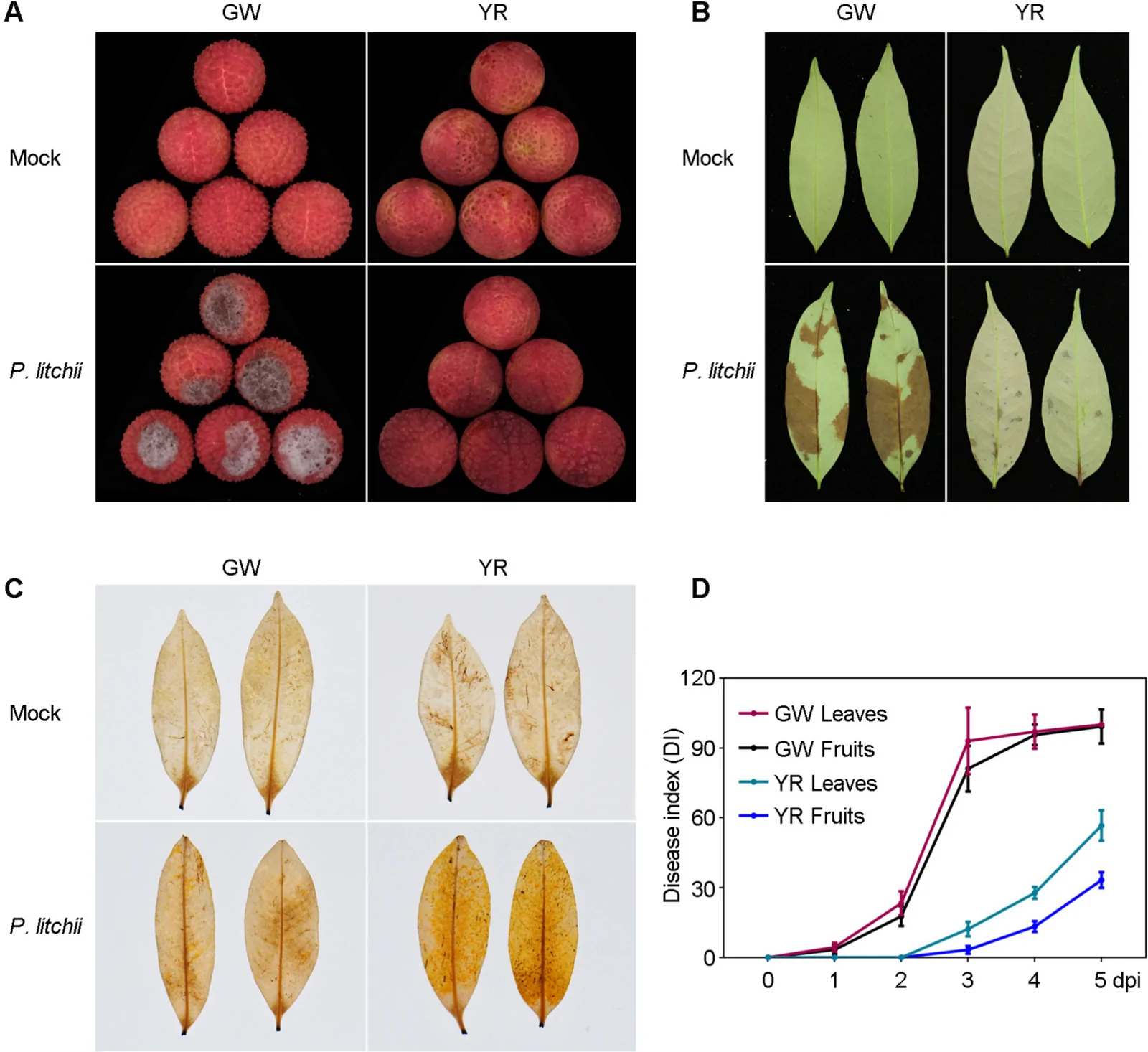
Analysis of the susceptibility of the selected varieties to LDB using GW and YR as examples. **A-B** Disease symptoms of GW and YR in (**A**) mature fruits and (**B**) leaves inoculated with *P. litchii* or Mock at 3 dpi. **C** Examination of H_2_O_2_ levels in leaves of GW and YR using DAB staining. The samples were inoculated with *P. litchii* or Mock and then stained at 24 hpi. **D** DI of GW and YR after inoculation. The data are presented as the means ± SD of three biological replicates

To verify the LDB sensitivity of the selected varieties, both the fruit and leaf tissues of YR and GW were inoculated with *P. litchii*. As shown in Fig. 2A, the pathogens grew rapidly, and the fruit surface was covered with masses of downy white sporangia and sporangiophores on GW, whereas no obvious changes were detected in YR at 3 dpostinoculation (dpi). Similarly, there was less necrosis in the YR leaves than in the GW leaves at 3 dpi (Fig. 2B). No significant differences were detected between the inoculated samples and the mock control samples. DAB staining to monitor the level of H_2_O_2_ in pathogen-inoculated leaves revealed that H_2_O_2_ accumulation increased in the infected plants (Fig. 2C). The growth of *P. litchii* indicated that the DI increased greatly in GW fruit and leaf tissues but changed slowly in YR at 3 dpi (Fig. 2D, Supplementary Fig. S2). These results indicate that compared with the LDB-sensitive GW variety, the YR variety exhibited LDB resistance, allowing for a comparative investigation of the internal resistance mechanisms of lychee against pathogen infection.

### Changes in internal metabolites in response to LDB infection in lychee

**Fig. 3:**
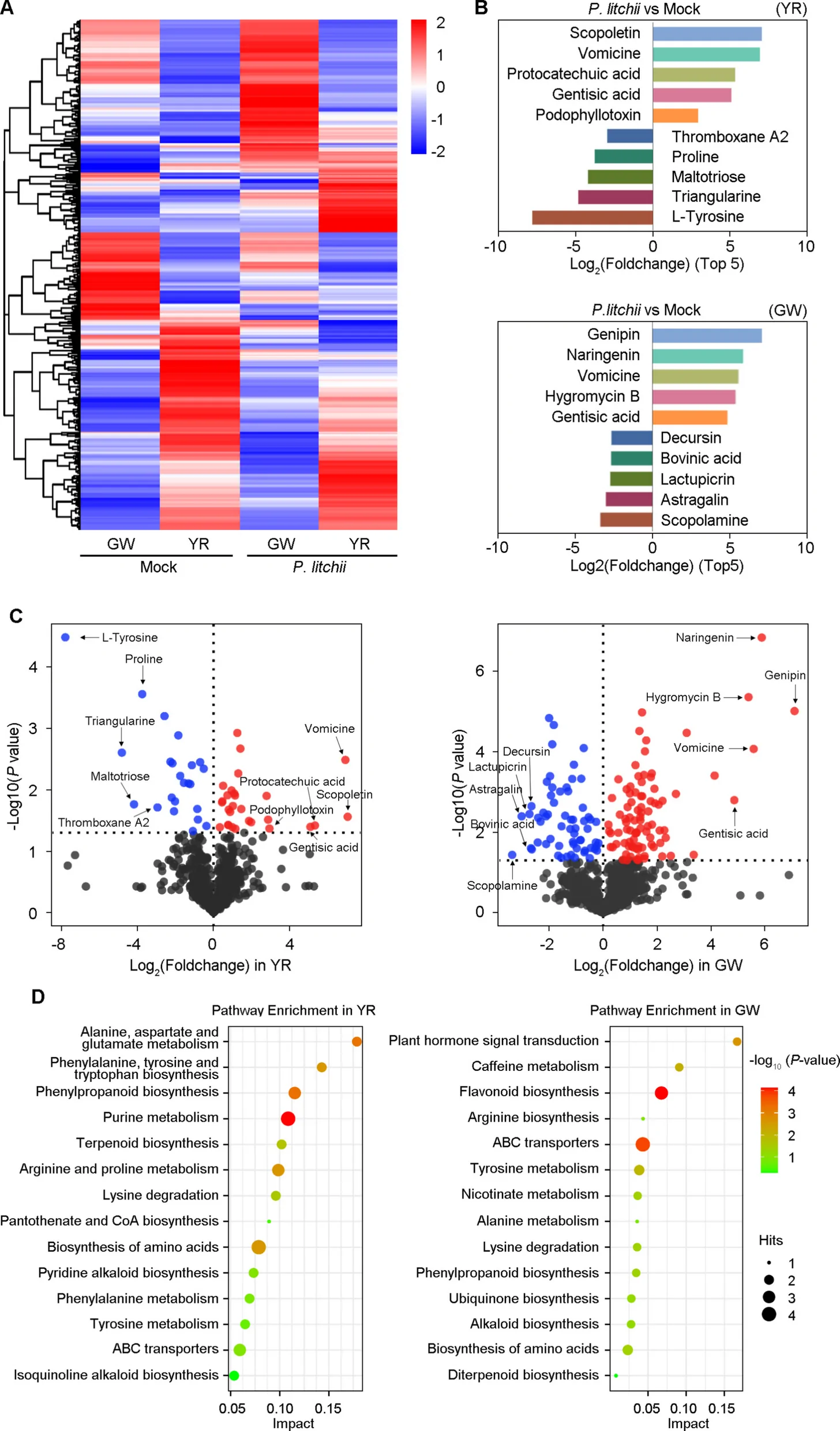
Metabolomic alterations in lychee stimulated with LDB. **A** Hierarchical clustering analysis of differentially abundant metabolites. The scale bar indicates the fold change. **B** The top 5 differentially abundant metabolites in YR and GW. **C** Volcano plot of differentially abundant metabolites in YR and GW. Each point represents a metabolite. Red, blue, and black spots indicate significantly upregulated, downregulated, and unchanged metabolites, respectively. **D** KEGG pathway analysis of differentially abundant metabolites

To explore the dynamic changes in the metabolites in response to LDB infection in lychee, widely targeted metabolomics was performed. Here, we used leaf tissues of the YR and GW varieties inoculated with *P. litchii* as examples for widely targeted metabolomics analysis. The differentially accumulated metabolites (DAMs) are shown in Fig. 3A (Supplementary Table S4). Notably, the five most abundant metabolites, scopoletin, vomicine, protocatechuic acid, gentisic acid, and podophyllotoxin PTOX (Fig. 3B), were significantly upregulated in YR, a disease-resistant lychee cultivar. In contrast, the levels of L-tyrosine, triangularine, maltotriose, proline, and thromboxane A2 decreased in YR leaves upon inoculation with *P. litchii* (Figs. 3B, C). In comparison, these metabolites exhibited pronounced fluctuations in the susceptible cultivar GW (Figs. 3B, C). Orthogonal partial least squares discrimination analysis (OPLS-DA) and correlation analysis revealed large differences between the metabolite profiles of the *P. litchii*-inoculated samples and those of the control (sterile water treatment as a mock) group (Supplementary Fig. S3). KEGG pathway and metabolomic analyses revealed that the majority of DAMs in YR were enriched in the phenylpropanoid biosynthesis pathway (Fig. 3D), suggesting that LDB-induced metabolites in lychee are predominantly derived from this defense-related pathway.

### Estimation of the ability of the candidate metabolite PTOX to inhibit *P. litchii* growth

**Fig. 4:**
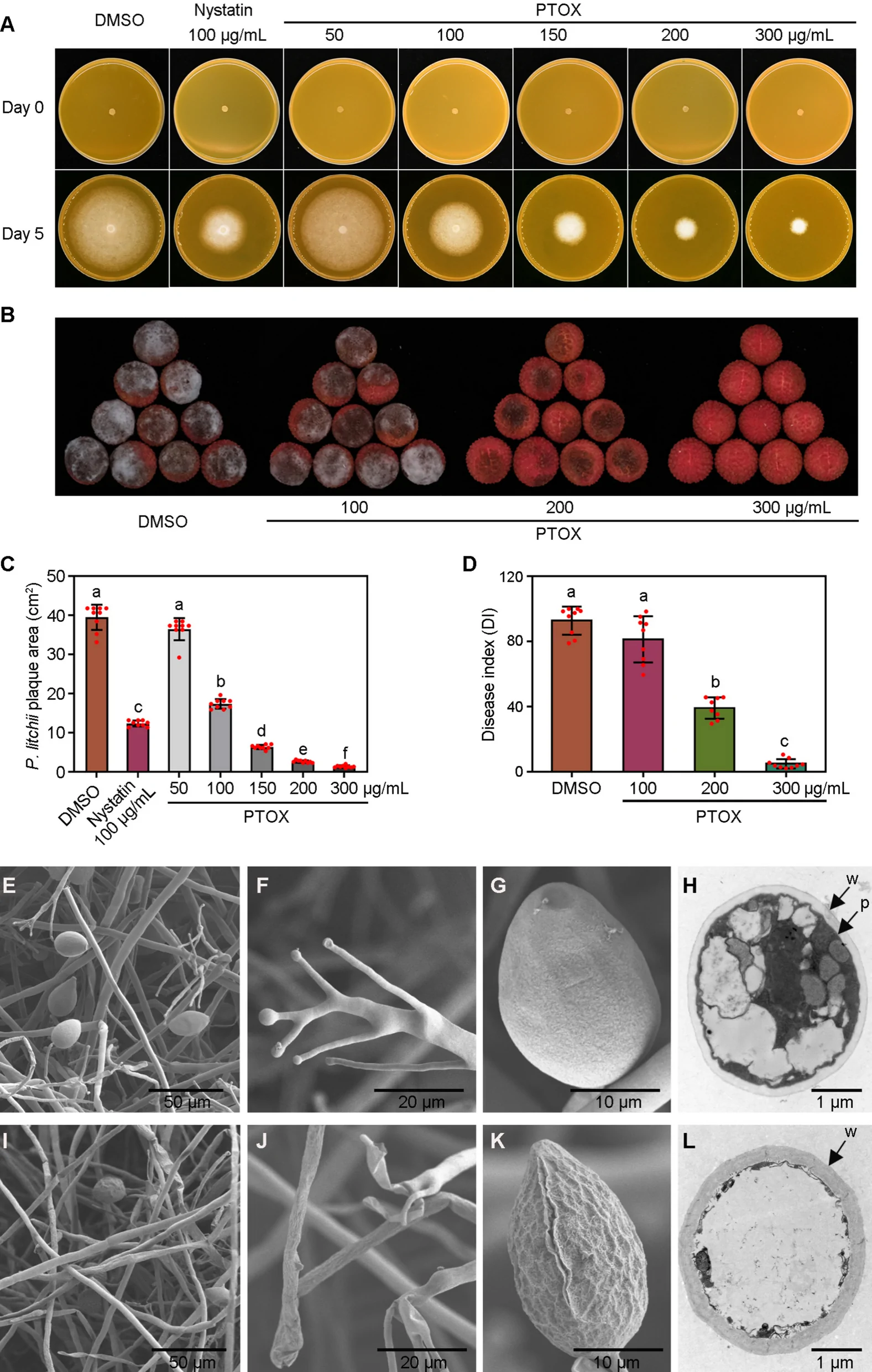
Estimation of the ability of PTOX to inhibit *P. litchii* growth. **A** In vitro examination revealed that PTOX inhibits the growth of *P. litchii* on CJA culture media. Nystatin was used as a positive control, and DMSO was used as a negative control. **B** PTOX inhibited the growth of *P. litchii* in the LDB-sensitive variety of GW in vivo. **C** Plaque area of *P. litchii* on CJA culture media at 5 dpi. **D** Disease index of GW fruit. The data are presented as the means ± SDs of three biological replicates. Lowercase letters indicate significant differences (*P* < 0.01). **E-L** SEM and TEM images of *P. litchii* growth under PTOX treatment. (**E–H**) represent the control group treated with DMSO, while (**I-L**) represent the treatment group receiving 200 μg/mL PTOX. The SEM images correspond to E–G and I-K, whereas the TEM images correspond to H and L. P, plasma membrane; W, cell wall

To validate the antifungal activities of these metabolites, we procured DAMs and individually assessed their inhibitory effects on *P. litchii*. Notably, PTOX, a lignan compound previously identified in diverse plant species (Cui et al. 2020), was unexpectedly detected among the DEGs of the disease-resistant cultivar YR. This finding represents the first report of PTOX in lychee. Our antifungal assays confirmed its potent inhibitory activity against *P. litchii*, representing a novel finding for this species. A series of concentrations of PTOX were added to CJA media, which were then inoculated with *P. litchi* for in vitro examination. Nystatin, a polyene–macrolide antifungal antibiotic against molds and yeasts, was used as a positive control (Melkoumov et al. 2013). As shown in Fig. 4A, the growth of *P. litchii* was inhibited by PTOX at concentrations greater than 100 μg mL^−1^ at 5 dpi, which resulted in inhibitory activity comparable to that of the positive control nystatin. The plaque size, indicated as the circle area for the growth of *P. litchii*, increased more slowly and was significantly lower in the PTOX-treated media than in the control media from 0 to 5 dpi (Fig. 4C, Supplementary Figs. S4 and S5). The inoculation of *P. litchi* on the LDB-sensitive variety of GW fruit together with PTOX treatment was further examined in vivo. Similar to the in vitro results, the application of PTOX significantly inhibited the growth of *P. litchi* (Fig. 4B), and the increase in DI in lychee fruit was apparently retarded (Fig. 4D).

To investigate the impact of PTOX on the growth of *P. litchii*, SEM images of sporangia and mycelia were obtained. In the control samples, the sporangia presented a typical, oval, and plump shape (Fig. 4E); the mycelia and sporangiophores presented a regular, linear, and consistent appearance (Figs. 4F, G). However, significant shrinkage and distortion were observed in the hyphae, and visible craters appeared on the surface of the sporangia after exposure to PTOX at a concentration of 200 μg mL^−1^ (Figs. 4I-K). These changes in morphology suggested that PTOX treatment may lead to damage to the subcellular structure of the hyphae and sporangia of *P. litchii*. TEM revealed a normal morphology of the cell wall, plasma membrane, vacuoles, and mitochondria in the sporangia of the control group (Fig. 4H). In comparison, PTOX treatment induced significant disorganization and coagulation of the cytoplasm, resulting in apparent damage to cytoplasmic organelles such as nuclei and mitochondria in the sporangia of *P. litchii* (Fig. 4L). The morphological and ultrastructural alterations observed in *P. litchii* demonstrated that the application of PTOX could efficiently suppress the growth of LDB in lychee. As a result, the internal lignan component PTOX may serve as an important substance for defending against pathogen infection induced by LDB in lychee.

### Chemical analysis to verify the candidate LDB-resistant metabolites

**Fig. 5:**
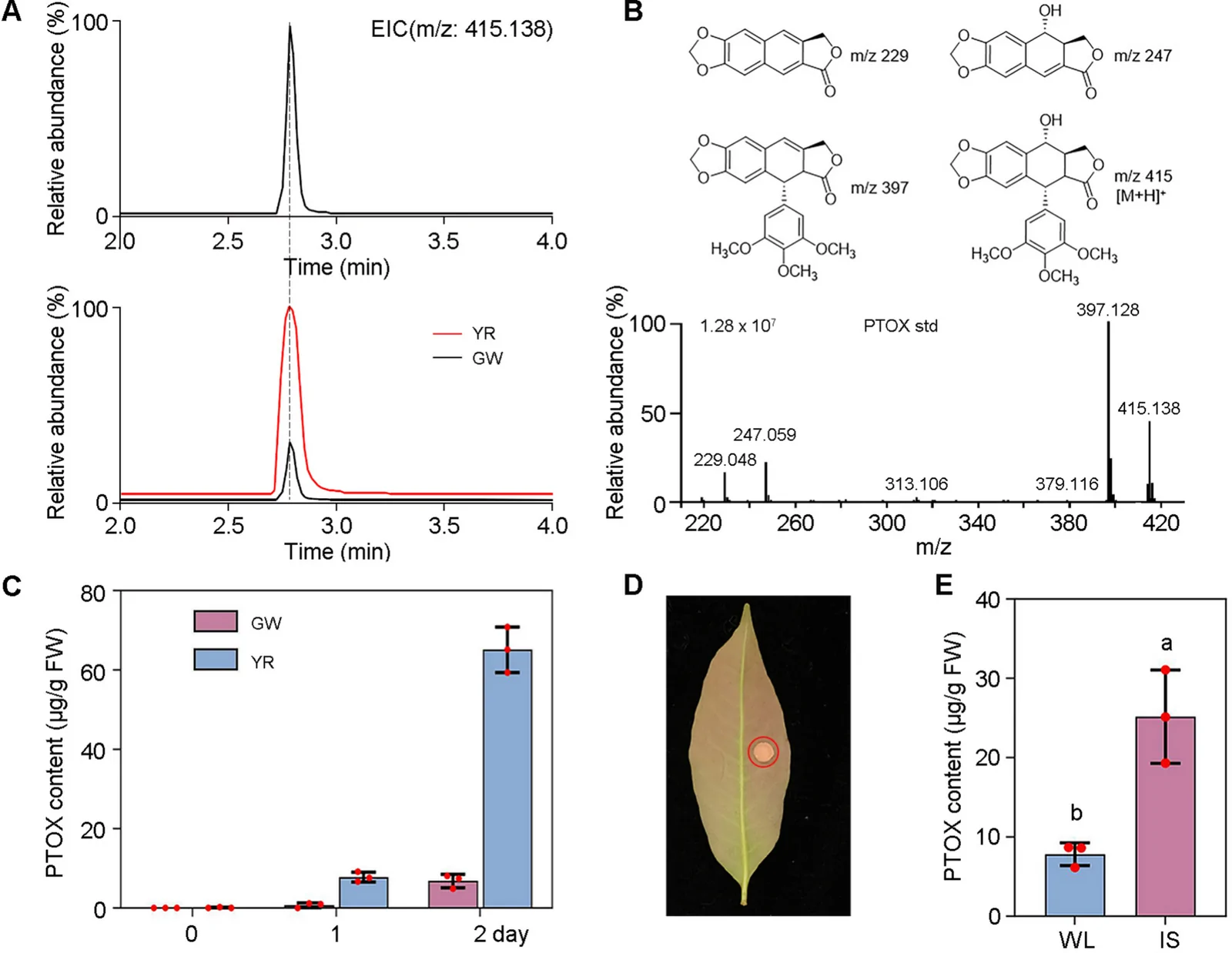
Chemical analysis of the lignan component of PTOX verified by UPLC–MS/MS in lychee. **A** Detection of PTOX metabolites in YR and GW. The upper panel shows the HPLC chromatogram of the PTOX standard, whereas the lower panel displays PTOX concentrations in YR and GW. **B** Characterization of the mass spectrum of the main m/z fragment of PTOX detected by UPLC–MS/MS analysis. **C** PTOX content in YR and GW lychee leaf tissues. **D-E** PTOX predominantly accumulated at pathogen stress sites. Schematic diagram of the treatment and sampling regions (**D**). Determination of PTOX content (**E**). WL, whole leaf; IS, inoculation site. The data are presented as the means ± SD of biological triplicates. Lowercase letters indicate significant differences (*P* < 0.01)

To verify the occurrence of PTOX and its differences among lychee varieties, chemical analyses of its quality and quantity were carried out using UPLC–QTOF–MS and UPLC–MS/MS, respectively. As shown in Fig. 5A, standard PTOX was detected with a retention time of 2.93 min, and similar peaks around this time were detected in both varieties, with a greater peak in YR leaf tissues than in GW leaf tissues (Fig. 5A). The mass spectra revealed a metabolite with the most prominent fragment at m/z 397.128, followed by the main structure at m/z 247.059 and m/z 229.048 and the fine molecular mass with [M + H]^+^ at m/z 415.138 (Fig. 5B, Supplementary Fig. S6). The mass spectra of the lychee samples were highly comparable to that of the PTOX standard, which verified the presence of PTOX in lychee. We further calculated the content of PTOX in lychee, which was significantly greater in the resistant variety YR than in the susceptible variety GW (Fig. 5C). We further found that the accumulation of PTOX increased around the inoculation sites (Figs. 5D, 4E). Considering the possible production of PTOX by microorganisms (Shah et al. 2021), sterile and nonsterile plants were cultivated and exposed to LDB (Supplementary Fig. S7A). The accumulation of PTOX increased in both types of leaves (Supplementary Fig. S7B), suggesting that the biosynthesis of PTOX might be stimulated by infection with LDB.

### Associations of transcriptional and metabolic analysis with the biosynthesis of target metabolites

**Fig. 6:**
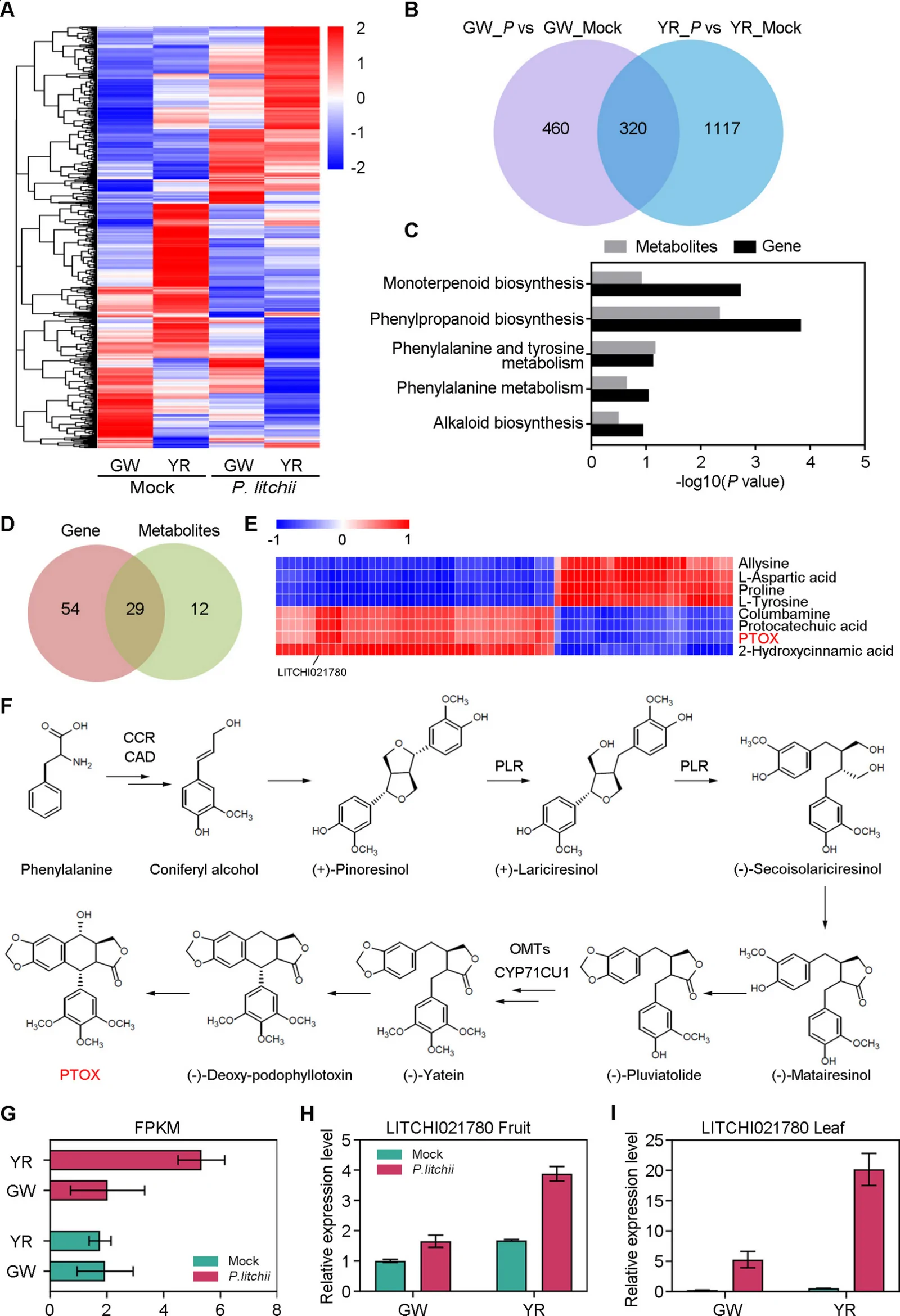
Combined transcriptomic and metabolomic analysis and gene expression data indicating the response of PTOX components to LDB stimuli in lychee. **A** Hierarchical clustering of RNA-seq data. **B** Venn diagrams showing the overlap of the DEGs. **C** Integrated analysis of changes in the transcriptome and metabolome. **D** Venn diagrams showing the overlap of the differentially enriched pathways in the transcriptome and metabolome analyses. **E** Heatmap of the correlations between the metabolome and transcriptome. **F** Overview of the biosynthesis of lignans, including PTOX, derived from the phenylpropanoid pathway. CCR, cinnamoyl CoA reductase; CAD, cinnamyl alcohol dehydrogenase; PLR, pinoresinol lariciresinol reductase; OMT, caffeic acid-O-methyltransferase. **G** FPKM values of YR and GW under *P. lithchii* stress. **H**-**I** Gene expression levels of key PTOX biosynthesis-related genes in (**H**) fruit and (**I**) leaf tissues, as validated by qRT‒PCR. The data are shown as the means ± SD from three biological replicates

RNA-seq analysis was carried out for the LDB-resistant YR and sensitive GW leaf tissues without or with inoculation of *P. litchii* at 1 dpi to elucidate the gene expression response to LDB infection (Fig. 6A, Supplementary Table S5). A total of 780 and 1437 DEGs were identified in GW_*P. litchii* versus GW_Mock and YR_*P. litchii* versus YR_Mock samples, respectively, and 320 DEGs overlapped between the two groups of samples (Fig. 6B, Supplementary Table S6). Among these DEGs, 503 in GW and 634 in YR were upregulated; 277 in GW and 803 in YR were downregulated (Supplementary Fig S8); and 320 genes were coregulated in both YR and GW (Fig. 6B, Supplementary Table S6). GO analysis revealed that most genes were involved in protein modification, oxidation reduction or transferase activity (Supplementary Fig. S9A; Supplementary Table S6). KEGG metabolism pathway analysis revealed that DEGs were largely enriched in secondary metabolite biosynthesis, plant–pathogen interactions, and terpenoid biosynthesis in both LDB-resistant YR and LDB-sensitive GW (Supplementary Fig. S9B, Supplementary Table S6).

Integrated analysis combining the transcriptome and metabolome profiles using KEGG enrichment data was performed. Among the 5 most enriched metabolic pathways, the most abundant responses of both DEGs and metabolites were located in the phenylpropanoid biosynthesis pathway (Fig. 6C). Among those differentially altered metabolic pathways, a total of 29 DEGs or metabolites were identified (Fig. 6D). Spearman correlation analysis of correlations between the transcriptome and metabolome results revealed that 40 DEGs were positively correlated and that 29 DEGs were negatively correlated with the phenylpropanoid pathway (Fig. 6E, Supplementary Table S7).

Given the observed accumulation of PTOX, a phenylpropanoid pathway derivative, in response to *P. litchi* infection (Fig. 5), we analyzed the genes associated with its biosynthesis. Multiple steps and enzymes were involved in PTOX biosynthesis through the phenylpropanoid pathway (Fig. 6F). To validate the genes involved in PTOX biosynthesis response to LDB stress, we quantified the relative expression levels of PTOX-related DEGs (Supplementary Fig. S10). Notably, FPKM analysis showed that *LITCHI021780* was constitutively expressed at significantly higher levels in the LDB-resistant YR cultivar than in the susceptible GW cultivar (Fig. 6G). This expression pattern was consistent in both fruit and leaf tissues (Figs. 6H, 6I). Functional characterization identified *LITCHI021780* as encoding an O-methyltransferase (OMT). This enzyme catalyzes two rate-limiting steps: the conversion of pinoresinol to secoisolariciresinol (Lau & Sattely 2015) and the methylation of (-)-pluviatolide to (-)-yatein (Ragamustari et al. 2013), both of which are essential precursors for PTOX production. These findings indicate that LITCHI021780 may function as a key regulatory gene in PTOX-mediated resistance to LDB. Consequently, we named LITCHI021780 as *LcOMT1*.

### Cloning and functional characterization of *LcOMT1*

**Fig. 7:**
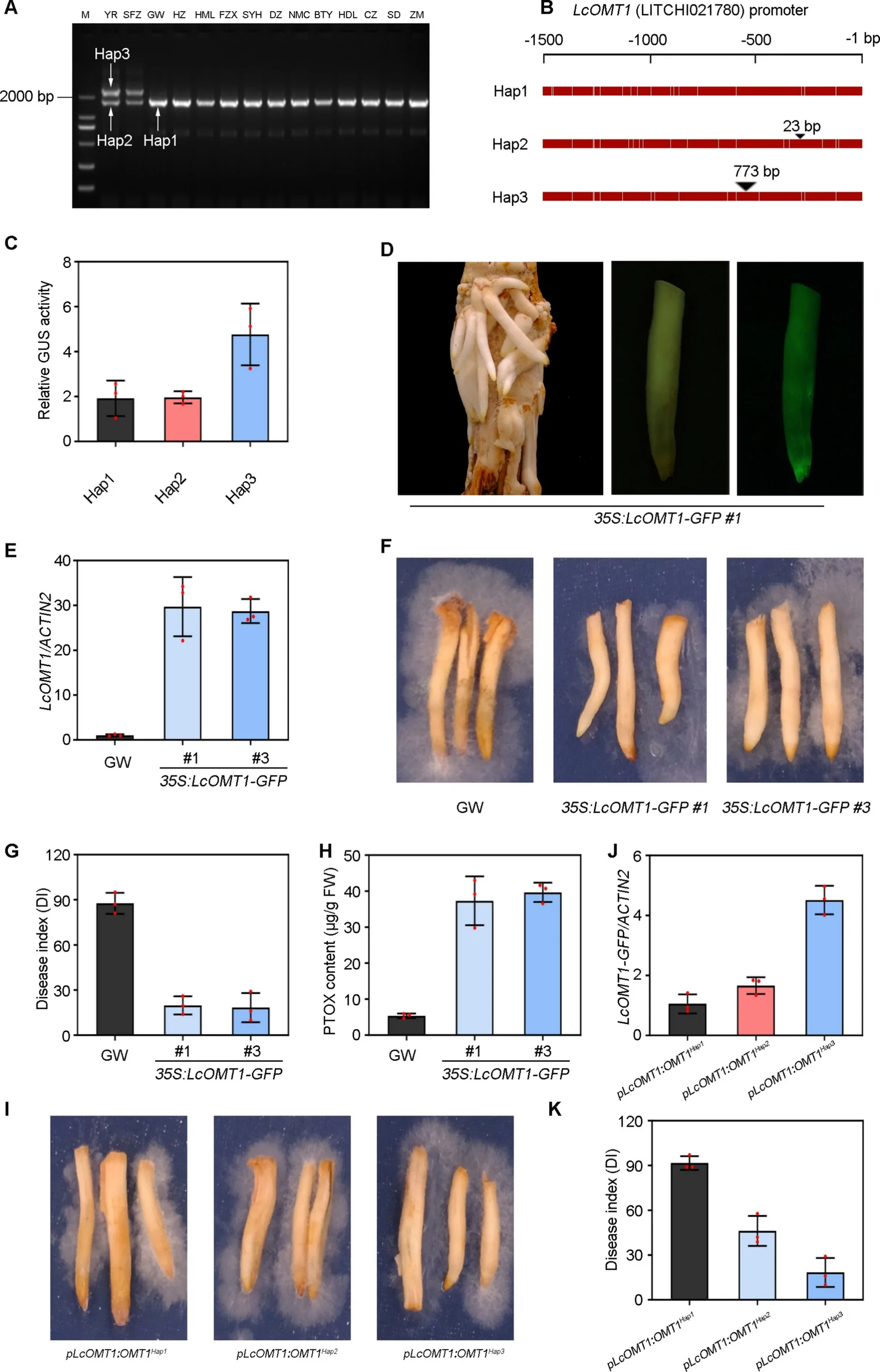
A large insertion in the *LcOMT1* promoter drives PTOX-mediated disease resistance. **A** Genetic analysis identified three distinct haplotypes (Hap1, Hap2, and Hap3) of *LcOMT1* within the lychee germplasm. Hap1 was uniquely detected in susceptible cultivars, whereas resistant cultivars specifically harbored Hap2 and Hap3, indicating their potential role in disease resistance. **B** Comparative structural analysis of *LcOMT1* haplotypes, highlighting sequence variations, particularly insertion mutations within the promoter region. **C** Functional characterization revealed that long-fragment insertion mutations significantly enhance the promoter activity of *LcOMT1*. **D** Establishment of an *Agrobacterium rhizogenes*-mediated genetic transformation system for *LcOMT1* overexpression in lychee hairy roots. **E** qRT‒PCR analysis of *LcOMT1* expression levels in transgenic hairy root lines, with GW lychee used as a control. **F** Phenotypic analysis demonstrated that the overexpression of *LcOMT1* significantly enhanced resistance against *P. litchii*, as evidenced by reduced lesion expansion. **G** Measurement of the DI in *LcOMT1*-overexpressing hairy roots. **H** Quantification of PTOX in *LcOMT1*-overexpressing hairy roots. **I** Functional validation revealed that, compared with Hap1 and Hap2, the Hap3 haplotype of *LcOMT1* conferred greater disease resistance in GW lychee hairy roots. **J** Comparative qRT‒PCR analysis of *LcOMT1* expression levels among transgenic hairy root lines harboring different haplotypes, revealing that Hap3 is the most highly expressed variant. **K** DI assessment of transgenic hairy roots with distinct *LcOMT1* haplotypes, confirming that Hap3 significantly reduced disease severity relative to Hap1 and Hap2. In C, E, G, H, J and K, the data are presented as the means ± SD from three biological replicates

To determine the functional role of *LcOMT1* in PTOX-mediated resistance to LDB, we cloned its sequence from two resistant and twelve susceptible lychee cultivars (Fig. 7A, Supplementary Fig. S11). Intriguingly, agarose gel electrophoresis of the PCR products revealed a single *LcOMT1* band in all the susceptible genotypes, whereas the resistant cultivar YR displayed two distinct bands (Fig. 7A, Supplementary Fig. S11). Sanger sequencing analysis revealed three distinct haplotypes (Hap1, Hap2, and Hap3), with susceptible cultivars exclusively harboring Hap1 and resistant cultivars showing heterozygosity for Hap2/Hap3. Promoter sequence analysis further revealed haplotype-specific insertions: Hap2 contained a 23 bp insertion, and Hap3 harbored a 773 bp large-fragment insertion in the *LcOMT1* promoter region (Fig. 7B). Sequence analysis revealed that the 773 bp sequence inserted in Hap3 contains several important disease resistance-responsive motifs, such as WRKYs (Supplementary Fig. S12). The promoter insertion mutations underlying the three distinct haplotypes prompted us to investigate the transcriptional activity of different *LcOMT1* haplotypes. Transient expression assays revealed that while both Hap1 and Hap2 induced GUS expression, Hap3 exhibited significantly greater promoter activity (Fig. 7C). The notably elevated promoter activity of Hap3 aligns with its exclusive occurrence in resistant cultivars (Fig. 7A). It indicated that enhanced *LcOMT1* expression mediated by this haplotype could promote PTOX biosynthesis to defend against *P. litchii* infection.

We subsequently generated *LcOMT1*-overexpressing transgenic hairy roots from the susceptible lychee cultivar GW using *Agrobacterium rhizogenes*-mediated transformation (Fig. 7D, Supplementary Fig. S13A). qRT-PCR analysis confirmed significantly greater *LcOMT1* expression in the transgenic lines than in the GW (Fig. 7E, Supplementary Fig. S13C). Following *P. litchii* inoculation, the transgenic roots presented increased LDB resistance, resulting in reduced lesion expansion (Fig. 7F, Supplementary Fig. S13B) and a lower DI (Fig. 7G). Additionally, the quantification of PTOX levels revealed a significant increase in the PTOX levels of the transgenic lines compared with those of the control (Fig. 7H, Supplementary Fig. S13D).

To delineate functional divergence among *LcOMT1* haplotypes, we generated transgenic hairy roots expressing individual haplotypes (Hap1, Hap2 and Hap3) in the susceptible cultivar GW (Fig. 7I). qRT-PCR analysis revealed that *LcOMT1* expression was significantly higher in the Hap3 transgenic lines than in the Hap1 and Hap2 lines (Fig. 7J). Pathogen challenge assays demonstrated that, compared with Hap1- and Hap2-expressing roots, Hap3-expressing roots presented greater resistance to *P. litchii* (Figs. 7I, K). These results indicate that Hap3, generated by a large-fragment insertion mutation, enhances *LcOMT1* transcription, thereby increasing PTOX content and enhancing lychee resistance to LDB.

## Discussion

Lychee is highly susceptible to LDB infection, the most serious disease affecting both plant growth and fruit quality at pre- and postharvest stages (Sun et al. 2019). However, germplasm with LDB resistance and the underlying regulatory mechanisms of LDB resistance remain poorly understood. In this study, we screened LDB-resistant varieties via population-scale antimicrobial analysis on the basis of lychee germplasm resources, and the responses of secondary metabolites to LDB resistance were subsequently identified and verified via metabolomics profiling and UPLC-MS/MS. The activity of the candidate lignan PTOX against LDB defense was verified both in vivo and in vitro. An insertion mutation in the *LcOMT1* promoter enhances PTOX biosynthesis by increasing transcriptional activity, consequently potentiating the innate resistance of lychee to LDB. Our study elucidates the regulatory mechanisms of LDB resistance in lychee across genetic and metabolic levels, which not only enhances our understanding of the molecular and biochemical basis of LDB resistance but also provides critical insights for breeding LDB-resistant lychee cultivars.

Current LDB control relies primarily on chemical fungicides, which pose significant risks to food safety, environmental integrity, and fungicide resistance development (Situ et al. 2023). This highlights the urgent need to characterize resistance metabolites and develop resistant cultivars. Although innate LDB resistance exists in some lychee germplasms, commercial breeding for LDB-resistant cultivars remains undeveloped. To address this, we screened a natural population of 288 geographically diverse lychee landraces for LDB-resistant variants (Fig. 1, Supplementary Table S1). Using standardized pathogen sensitivity assays (Sun et al. 2019), two-year consecutive testing confirmed stable resistant/susceptible phenotypes toward *P. litchii* growth across these accessions (Fig. 2). Germplasm resources are indispensable in agricultural research because of their biodiversity, enabling the discovery and utilization of elite genetic resources while avoiding challenges associated with genetically modified materials (Anumalla et al. 2015; Dar et al. 2015). The availability of lychee germplasms thus enables comprehensive investigation of LDB resistance mechanisms through large-scale screening. Furthermore, the LDB-resistant germplasm identified via this screening provides valuable genetic material and a theoretical basis for breeding LDB-resistant cultivars.

Plants generally recognize invasion signals and produce resistance factors to initiate defense responses against pathogen infection and insect infestation (Eyles et al. 2010). To screen for significantly differential metabolites among the selected comparable varieties, we performed widely targeted metabolomics analysis. This approach utilizes a comprehensive metabolite library with broad coverage of plant compounds, which greatly facilitates the identification of differential metabolites and enhances the accuracy and reliability of metabolite annotation (Sawada et al. 2009). This strategy helps to rapidly narrow the selection range and characterize unknown potential response metabolites aligned with our study objectives. Notably, the top DAMs, such as gentisic acid, protocatechuic acid, scopoletin, and PTOX, were highly enriched in plants (Fig. 3). Interestingly, the differentially accumulated lignan PTOX was newly identified in lychee. In addition, consistent with the significant changes detected via metabolomics analysis, we further carefully verified the target metabolites such as PTOX via UPLC-QqQ-MS/MS by comparison with the standard compound in detailed spectral analysis. It provides validated, solid evidence that goes beyond the limitations of replication studies, showing that PTOX naturally accumulates in lychee plant tissues (Fig. 5, Supplementary Figure S6). PTOX is an aryltetralin-type lignan compound that was originally discovered in the plant *Podophyllum peltatum* and has also been detected in other plant species (Shen et al. 2022). As a diphenolic compound derived from the phenylpropanoid pathway, PTOX has potential anticancer, antiviral and antioxidant activities (Ardalani et al. 2017; Cui et al. 2020). Despite its antiviral and antimicrobial activity, the ability of PTOX to protect against fungal pathogens or inhibit the growth of *P. litchii* is still uncertain (Pagare et al. 2015). We further tested the LDB resistance of PTOX both in vitro and in vivo. Consistent with the general screening results, the PTOX metabolite displayed the capacity to significantly inhibit the growth of LDB on CJA media or on fresh lychee (Fig. 4). In addition, the accumulation of secondary metabolites might modulate the efficiency of pathogen defense (Sweelam et al., 2018). Notably, the resistant cultivar YR accumulated markedly higher levels of PTOX than susceptible varieties (Fig. 5). These results suggest that the biosynthesis and accumulation of PTOX in lychee might be one of the key factors involved in plant immunity against LDB infection. Accordingly, PTOX, a lignan, might be an important active substance conferring LDB resistance, and its role was functionally validated for the first time in lychee.

Multiomics analysis provides a useful strategy for revealing the key factors investigated from the detected data profiles (Muhammad et al. 2023). The transcriptional analysis of gene expression further revealed that the DEGs involved in phenylpropanoid biosynthesis were enriched in one of the pathways most strongly affected by LDB infection (Fig. 6). The biosynthesis of PTOX involves the catalysis of various enzymes, such as OMTs (Lau and Sattely 2015; Shen et al. 2022). Specifically, OMT methylates phenolic hydroxyl groups on pathway intermediates. Unmethylated precursors exhibit substantially reduced or incomplete biological activity, whereas OMT-mediated methylation confers structural stability, enabling PTOX to effectively bind to cellular targets and exert its pharmacological effects (Chen et al. 2011; Lau and Sattely 2015; Xia et al. 2000). Furthermore, OMTs functionally coordinate with upstream enzymes such as phenylalanine ammonia-lyase (PAL) and 4-coumarate-CoA ligase (4CL) in the phenylpropanoid pathway (Lau and Sattely 2015). Fluctuations in the activity of these upstream enzymes directly modulate precursor availability, thereby indirectly influencing OMT catalytic efficiency. Our study revealed three *LcOMT1* haplotypes in lychee germplasms with differential LDB responses. Exploiting the inherent heterozygosity of lychee (Hu et al. 2022), resistant cultivars harbor a heterozygous *LcOMT1* genotype comprising Hap2 and Hap3. A long-fragment insertion in the promoter region augments transcriptional activity, driving elevated PTOX biosynthesis and conferring enhanced LDB resistance. This mechanistic insight directly explains the observed PTOX accumulation in resistant lines and pathogen-induced PTOX upregulation upon *P. litchii* inoculation.

**Fig. 8:**
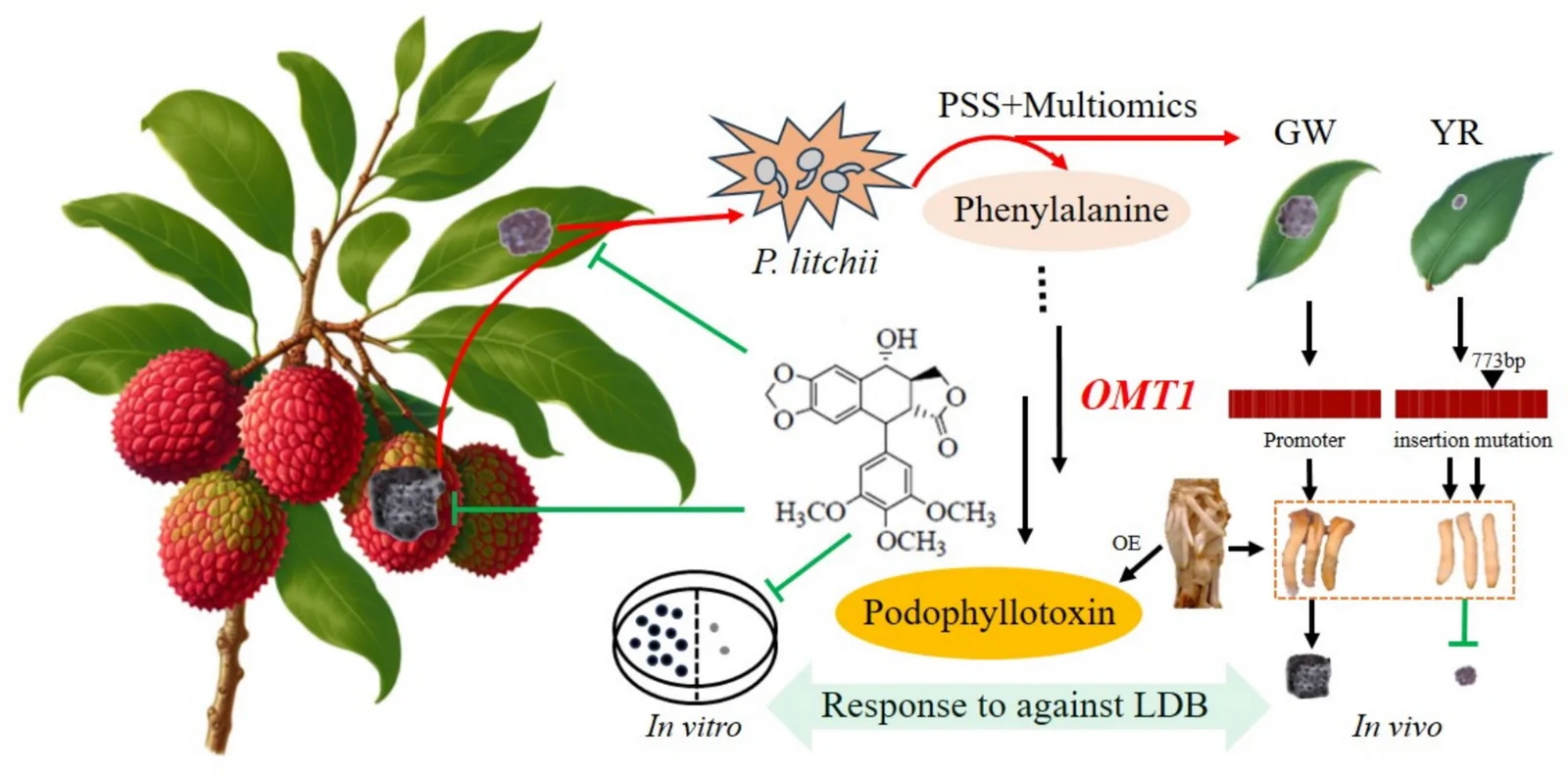
A schematic of working model elucidating the intrinsic response of lignin PTOX to protect against LDB infection in litchi by both in vivo and in vitro examinations. PSS, population-scale screening; GW, Guiwei; YR, Yurong; LDB, lychee downy blight. The graph is not drawn in scale

Biologically friendly control strategies represent a cornerstone of sustainable agricultural systems. Plant-derived natural products have emerged as promising eco-friendly biofungicide candidates (Khursheed et al. 2022), and PTOX derivatives have already been deployed in crop protection as registered botanical pesticides (Sun et al. 2017). In this study, we identified lychee-derived PTOX as a novel antifungal aryltetralin lignan, and experimentally validated its inhibitory efficacy against *P. litchi* both in vivo and in vitro (Fig. 8). These findings establish PTOX as an endogenous defense metabolite in lychee and highlight its translational potential as a sustainable bioactive scaffold for next-generation biopesticide formulations.

## Conclusion

In this study, the molecular mechanisms underlying lychee resistance to LDB are systematically elucidated through large-scale germplasm screening and multiomics analysis. An evaluation of 288 global lychee accessions identified the highly resistant cultivar YR and the susceptible cultivar GW, enabling the comparative study. Widely targeted metabolomics revealed the marked accumulation of PTOX, a previously undocumented antifungal lignan in lychee, in the resistant variety of YR. Functional characterization confirmed the potent inhibitory effects of PTOX on *P. litchii*, which disrupted hyphal growth and sporangial development through severe ultrastructural damage. *LcOMT1*, encoding a key OMT involved in phenylpropanoid metabolism, was identified as a central genetic determinant of resistance to *P. litchii*. Resistant genotypes carry a 773-bp insertion (Hap3 haplotype) in the *LcOMT*1 promoter that amplifies its transcriptional activity. In addition, we established a lychee hairy root transformation system in which the overexpression of *LcOMT1* or Hap3 induced the upregulation of PTOX biosynthesis and enhanced the LDB defense. These results provide new insight into the causal link between the Hap3-*LcOMT1*-PTOX module and disease resistance in lychee. This study not only reveals the genetic-metabolic regulatory network of lychee resistance to LDB but also provides a theoretical basis for developing PTOX-based biofungicides and breeding LDB-resistant lychee varieties.

## Materials and methods

### Plant materials

Lychee plant materials were obtained from the National Lychee Germplasm Repository, Institute of Fruit Tree Research, Guangdong Academy of Agricultural Sciences, Guangzhou, China. In this study, both leaf and fruit tissues were collected from 288 lychee landraces from different geographic areas (Supplementary Table S1). Fresh leaf and fruit samples were packed into 0.03 mm bags and immediately transported to the laboratory within 2 h. The mature leaves and ripe fruits of uniform shape and color and fine intact samples without any defects were carefully selected for further experiments. The process of cultivating sterile lychee seedlings was meticulously carried out. Briefly, mature fruit seeds were subjected to a rigorous disinfection procedure with a 0.1% mercuric chloride solution for 15 min and rinsed with sterile water to remove any residual traces. The seeds were then cultivated on autoclaved MS culture media at 27 °C.

### Inoculation of pathogens to evaluate the capacity of LDB-resistant bacteria

To test susceptibility to LDB in lychee, the pathogen *P. litchii* was isolated from deteriorated fruit and cultured on carrot juice agar (CJA) media at 27 °C in the dark (Liu et al. 2023). The spore suspension was subsequently adjusted to 4 × 10^4^ spores/mL by sterilizing with water for inoculation. The fruit and leaf tissues were randomly divided into three groups as replications, each consisting of 10 samples. All fruits and leaves were inoculated with 5 µL of a sporangial suspension of *P. litchii* and subsequently transferred to an incubator at 27 °C.

Pathogenicity assays were performed as previously described with minor modifications (Sun et al. 2019). The disease index (DI) was evaluated at 3 days postinoculation (dpi) with the following scale: 0 for no disease, 1 for lesion areas less than 25%, 2 for lesion areas ranging from 25 to 50%, 3 for lesion areas ranging from 50 to 75%, and 4 for lesion areas greater than 75%.

### Histochemical analysis

The 3,3'-diaminobenzidine (DAB) staining method was used to examine the accumulation of H_2_O_2_ in the samples (Liu et al. 2021). After the leaf tissues were inoculated with *P. litchii* or mock (sterile water) after 24 h postinoculation (hpi), the samples were transferred to the staining solution (1 mg mL^−1^ DAB, Sigma) for 12 h. The stained leaf samples were then decolorized in destaining solution (70% ethanol and 30% ethanoic acid), and images were then acquired under a stereoscope (Leica M165C).

### Scanning electron microscopy (SEM) and transmission electron microscopy (TEM)

The impact of PTOX on the ultrastructure of *P. litchii* was investigated through SEM and TEM observations. The treatment and sample preparation with PTOX followed the protocol outlined in a previous report (Xu et al. 2018). The samples for SEM analysis were subsequently examined via a scanning electron microscope (Hitachi, SU8100) operating at 5 kV, whereas the samples for TEM analysis were observed with a transmission electron microscope (Hitachi, HT7800) operating at 80 kV.

### Metabolites extraction for widely targeted metabolomics analysis

The fresh samples about 100 mg were transferred to a 2 mL eppendorf tubes and suspended with 500 μL extract solvent (methanol:water, 1:1, V/V). After vortex for 30 s, the samples were homogenized at 35 Hz for 4 min, and sonicated 15 min with ice-water bath. Then the samples were extracted over night at 4 ℃ on a shaker. Then centrifuged at 12,000 rpm for 15 min at 4 ℃. The supernatant was carefully filtered through a 0.22 μm microporous membrane and transferred to 2 mL glass vials, and take 30 uL from each sample and pooled as QC samples. Finally, 50 μL supernatant was transferred to LC vials for subsequent liquid chromatography-mass spectrometry (LC–MS) analysis (Allwegene Technology Co., Ltd, Beijing, China).

### UHPLC-MS–MS detection and data analysis

Ultra-high-performance liquid chromatography (UPLC) separation was performed using an EXIONLC LC System (Sciex) equipped with a Waters Acquity UPLC HSS T3 column (1.8 μm, 2.1 × 100 mm). Mobile phase A consisted of 0.1% formic acid in water, and the mobile phase B consisted of 100% acetonitrile. The following gradient program was used: 98% B, 0.5 min; 98%−50% B, 0.5–10 min; 50%−5% B, 10–11 min; 5% B, 13 min; 5% −98% B, 13–13.1 min; and 95% B, 15 min. The flow rate was 0.5 mL/min, the injection volume was 2 μL, and the autosampler was maintained at 4 °C. For metabolite detection, a Sciex QTRAP 6500 + mass spectrometer (SCIEX) was applied for assay development. Typical ion source parameters were: IonSpray Voltage: + 5500/−4500 V, Curtain Gas: 35 psi, Temperature: 400 °C, Ion Source Gas 1:60 psi, Ion Source Gas 2: 60 psi, DP: ± 100 V.

After initial method development, widely targeted metabolomics analysis was performed using a house library containing more than 5000 authentic reference standards. This library covers a comprehensive spectrum of plant metabolite classes, including phenylpropanoids (19%), flavonoids (13%), other secondary metabolites (14%), alkaloids (8%), sugars and glycosides (6%), amino acids and peptides (5%), as well as other classes each comprising less than 5% e.g., phenolic acids, benzoic acids, alcohols, nucleic acids, polyketides, quinones, and lignans. A small fraction of metabolites (12%) remains unclassified. This platform combines the broad metabolite coverage characteristic of untargeted metabolomics with the high sensitivity and precise quantification capability of targeted metabolomics. Using UHPLC-QqQ-MS/MS in multiple reaction monitoring (MRM) mode, this approach enables the simultaneous identification and quantification of thousands of metabolites in a single analysis.

SCIEX Analyst Work Station Software (Version 1.6.3) was employed for MRM data acquisition and processing. In-house R program and database were applied to peak detection and annotation. A series of quality control steps were applied. Metabolite features detected in less than 20% of experimental samples or in less than 50% of QC samples were removed from data analysis. Missing values in the raw data were then imputed using half of the minimum value. An internal standard normalization method was employed. Finally, features with a relative standard deviation (RSD) > 30% were excluded from further analysis. The resulting three-dimensional data including peak number, sample name, and normalized peak area were imported into the R package metaX for principal component analysis (PCA) and orthogonal partial least squares discriminant analysis (OPLS-DA). The resulted three-dimensional data involving the peak number, sample name, and normalized peak area were fed to R package metaX for principal component analysis (PCA) and orthogonal partial least squares discriminant analysis (OPLS-DA). To refine the analysis, the variable importance in projection (VIP) values from the first principal component were obtained. VIP values summarize the contribution of each variable to the model. Metabolites with VIP > 1 and *P* < 0.05 (Student's *t-*test) were considered significantly differentially accumulated metabolites (DAMs). Metabolic pathways were annotated using public databases including KEGG (http://www.kegg.jp) and MetaboAnalyst (http://www.metaboanalyst.ca/). The metabolomics raw data have been deposited in the OMIX database (China National Center for Bioinformation) under accession number OMIX016396.

### Verification and quantification of PTOX in lychee

To further verify the significantly different secondary metabolites, the PTOX substances were qualified and quantified again. Approximately 100 mg of fresh sample was ground in liquid nitrogen and dissolved in 1 mL of cold methanol. The mixtures were kept at −20 °C for 2 h and then centrifuged at 15,000 × g for 15 min at 4 °C. The supernatants (500 µL) were collected and mixed with 500 µL of water. The samples were filtered through a 0.22 μm nylon membrane and analyzed with an ultra-high performance liquid chromatography–quadrupole time-of-flight mass spectrometry (UPLC-QTOF-MS) system consisting of an ACQUITY UPLC I-Class (Waters, Milford, MA, USA) and Xevo® GS2 QTOF (Waters, Milford, MA, USA) and equipped with an HSS T3 column (1.8 μm, 100 × 2.1 mm, Waters Corp, Milford, MA) at 40 °C. Data were collected for the mass range of 50–1200 Da in MSE mode and analyzed using MassLynx™ software (Waters Corporation). The characteristic ion for PTOX was m/z 415.1393.

### Assays of the antimicrobial activity of target metabolites

The LDB resistance of the differentiated metabolites was tested in vitro to further verify their response to LDB infection in lychee. Nystatin is a polyene–macrolide antifungal antibiotic that is widely employed as an antifungal agent against molds and yeasts, and it was used as a positive control in this study (Melkoumov et al. 2013). Nystatin (GlpBio, Cat^#^ GC10090) or PTOX (GlpBio, Cat^#^ GC10521) was dissolved in DMSO (GlpBio, Cat^#^ GK12002). DMSO was used as the negative control. Different concentrations of PTOX were prepared as test samples. The tests included both in vitro culture of CJA medium and in vivo inoculation on fruit surfaces. Mycelial plugs (5 mm in diameter) of *P. litchii* were inoculated into each medium and group of fruits and stored at 27 °C. The changes in the fungal colonies were continuously measured and calculated from the day of inoculation (designated day 0) to day 5. Three biological replicates were used for each experiment. The growth of *P. litchii* in each experimental and control group was randomly selected and photographed, and the growth of *P. litchii* was calculated as the area colonized on the media, and the DI mentioned above was calculated.

### RNA-Seq analysis

The leaf tissues without or with *P. litchii* at 24 hpi were collected for total RNA extraction using the FastPure Plant Total RNA Isolation Kit (Vazyme, Cat^#^ RC401-01). RNA sequencing was performed by Allwegene Technology Inc. (Beijing, China) via the Illumina NovaSeq platform, resulting in 23–33 million reads per sample. All the downstream analyses were based on clean high-quality data mapped to the lychee reference genome (Hu et al. 2022). Differential expression analysis of two conditions/groups (three biological replicates per condition) was performed using the DESeq R package (1.18.0). DESeq provides statistical routines for determining differential expression, and the resulting *P* values were adjusted using the Benjamini and Hochberg’s approach for controlling the false discovery rate. Genes with an adjusted *P*-value < 0.05 and a fold change > 2 according to DESeq were considered differentially expressed. The gene expression patterns are graphically represented in a heatmap. Gene Ontology (GO) enrichment analysis of the differentially expressed genes (DEGs) was implemented with the GOseq R package. KEGG analysis was performed via KOBAS software to test the statistical enrichment of DEGs in KEGG pathways (Mao et al. 2005). The RNA-seq data were deposited in the NCBI Gene Expression Omnibus database under accession number GSE201243.

### qRT-PCR Analysis

Total RNA was extracted using a plant RNA Kit (Vazyme, Cat^#^ RC401-01). Quantitative RT‒PCR was performed using SYBR Green Master Mix (Vazyme, Cat^#^ Q711-02) on a LightCycler 480 Thermal Cycler (Roche) as described previously. Lychee ACTIN was used as an internal control for sample normalization during real-time RT-PCR analysis (Sun et al. 2019). The primers used are listed in Supplementary Table S2. The relative expression of genes was expressed according to the qRT‒PCR calculation method (Schmittgen and Livak 2008). Three independent biological replicates for each gene of each sample were performed.

### Haplotype analysis of *LcOMT1* in lychee

To characterize the haplotypes of *LcOMT1*, gene-specific primers (Supplementary Table S2) were designed to clone the full-length sequences of the CDS, promoter, and genomic DNA from diverse lychee cultivars. The PCR products were purified and sequenced, followed by multiple sequence alignment via the MUSCLE algorithm in MEGA11 to identify single-nucleotide polymorphisms (SNPs) and insertion/deletion variations (InDels).

### Plasmid Construction

For the three haplotype-specific promoter constructs, *LcOMT1* promoter fragments of Hap1, Hap2, and Hap3 amplified from GW and YR cultivars were cloned and inserted into the *pHY107* vector containing a β-glucuronidase (GUS) reporter. For the *p35S:OMT1-GFP* and *p35S:OMT1-RUBY* constructs, the coding regions of *OMT1* without the stop codon were amplified from YR and cloned and inserted into the modified vector *p35S-GFP* or *p35S-RUBY* with the *pDOE* backbone (Yin et al. 2025).

For the construction of the *pOMT1:OMT1*^*Hap1*^, *pOMT1:OMT1*^*Hap2*^ and *pOMT1:OMT1*^*Hap3*^ constructs, the full-length genomic sequences of the *LcOMT1* haplotypes were individually amplified from the genomic DNA of the GW and YR lychee cultivars using high-fidelity PCR. The amplified fragments, encompassing the native promoter and coding region of *LcOMT1*, were cloned and inserted into the *pDOE* vector, replacing the 35S promoter region. This strategy allows the expression of each haplotype under its endogenous regulatory elements. The recombinant plasmids were verified by restriction enzyme digestion mapping and Sanger sequencing. All primers used for vector construction are listed in Supplementary Table S2.

### Transient expression assay and enzyme activity detection

A *p35S:LUC* construct (encoding firefly luciferase) was coinfiltrated as an internal control to normalize the transient expression efficiency in *N. benthamiana* leaves. *Agrobacterium tumefaciens* strain GV3101 harboring the respective constructs was resuspended in infiltration buffer (10 mM MES, 10 mM MgCl₂, 150 μM acetosyringone, pH 5.6) to an OD₆₀₀ of 0.5 (Liu et al. 2022). Leaves of 4-week-old plants were infiltrated using a 1 mL needleless syringe, and samples were collected 48 h postinfiltration for enzyme activity assays, following the method described by Liu et al. (2021). Relative GUS activity was calculated by normalizing GUS activity against that of LUC, and the data are presented as averages of three biological replicates. All primers used for the transient expression assay are listed in Supplementary Table S2.

### Genetic transformation of lychee hairy roots

To generate transgenic hairy roots from lychee, we employed the *Agrobacterium rhizogenes*-mediated transformation method described by Yin et al. (2025). Briefly, a 2-cm vertical wound was made on healthy branches using a grafting knife. The wound was then inoculated with *Agrobacterium rhizogenes* strain MSU 440 harboring the target expression vector. The inoculated site was immediately sealed with sterile cling film and wrapped with aluminum foil for protection from light. Hairy root primordia emerged from the wound 4–8 weeks after inoculation.

### Statistical analysis

On the basis of the gene expression and metabolite content data, Pearson correlation tests were used to detect associations between discriminant gene expression and discriminant metabolite content. DEGs (*P* < 0.05 and fold change > 2) and SDMs (VIP > 1 and *P* < 0.05) were used for the integrative analysis. DEGs and differentially abundant metabolites were mapped to the KEGG pathway database to obtain their common pathway information. All the statistical analyses and graph constructions were performed using GraphPad Prism 9 (GraphPad Software Inc., La Jolla, CA, USA) or R Language (R version 4.2.3, psych and ggplot2 packages). Statistical differences were determined via one-way ANOVA, with significant differences at *P* < 0.05.

## Supplementary Information


Supplementary Material 1. Fig S1. LDB is one of the major diseases in lychee. Fig S2. GW and YR displayed stable susceptibility and high resistance against LDB, respectively. Fig S3. Orthogonal partial least squares-discriminant analysis (OPLS‑DA) of widely targeted metabolomics profiles among different sample groups. Fig S4. PTOX inhibits the mycelial growth of *P. litchii* on CJA medium. Fig S5. Plaque areas of *P. litchii* on CJA medium supplemented with different concentrations of PTOX. Fig S6. Mass spectral analysis and identification of PTOX in YR and GW lychee. Fig S7. Determination of PTOX content in naturally grown leaves and axenic seedling leaves following LDB treatment. Fig S8. Venn diagram showing the number and overlap of DEGs. Fig S9. Functional enrichment analysis of DEGs in YR vs. GW under LDB stimulation. Fig S10. Gene expression analysis of PTOX biosynthesis genes in lychee fruits (A) and leaves (B). Fig S11. Evaluation of LDB resistance in different lychee cultivars. Fig S12. Alignment of *LcOMT1* promoter sequences. Fig S13. Overexpression of *LcOMT1* enhances GW resistance to LDB. Supplementary Material 2. Table S1. List of lychee cultivar names.Supplementary Material 3. Table S2. Sequence of primers used in this study.Supplementary Material 4. Table S3. Descriptive statistics and ANOVA analysis of DIs in 288 lychee accessions.Supplementary Material 5. Table S4. Widely targeted metabolomics analysis of LDB-resistant (YR) and LDB-susceptible (GW) lychee.Supplementary Material 6. Table S5. Quality control of transcriptomes from GW and YR leaves under Mock (sterile water) or LDB treatment.Supplementary Material 7. Table S6. Differentially expressed genes in GW and YR.Supplementary Material 8. Table S7. The DEGs were investigated by integrating the transcriptome and metabolome data. 

## Data Availability

All relevant data are within the paper and supplementary files.

## Abbreviations

DAMs: Differentially accumulated metabolites
DEGs: Differentially expressed genes
DI: Disease index
GW: Guiwei
LDB: Lychee downy blight
OMT: O-methyltransferase
PTOX: Podophyllotoxin
YR: Yurong
