# Supplementary material for: Deciphering podophyllotoxin-mediated defense against downy blight in Litchi chinensis Sonn. via integrated metabolomics and transcriptomics
Source: Mol Hortic. 2026 Sep 2;6:63. doi: 10.1186/s43897-026-00257-4 (PMC13536555; doi:10.1186/s43897-026-00257-4)
Supplement: Supplementary file 1 [file 43897_2026_257_MOESM1_ESM.docx]

**Supplementary Fig S1. LDB is one of the major diseases in lychee.**

The disease causes severe damage to (**A**) lychee leaves, (**B)** nearly mature fruits, and (**C**) fully mature lychee fruits. Photographs were taken at the National Lychee Germplasm Repository, Guangzhou, China.

**Supplementary Fig S2. GW exhibited stable susceptibility, while YR showed high resistance to LDB.**

DIs of fresh fruits (**A**) and fresh leaves (**B**) were evaluated in 2021 and 2022 to assess LDB resistance. Data are means ± SD of three biological replicates.

**Supplementary Fig S3. Orthogonal partial least squares-discriminant analysis (OPLS‑DA) of widely targeted metabolomics profiles among different sample groups.**

(**A**-**B**) Score plots of OPLS‑DA models for different comparison groups. The X‑axis (T score) represents the score of the first predictive principal component, and the y‑axis (Orthogonal T score) represents the orthogonal principal component score. Different symbols and colors indicate distinct sample groups.

(**C**-**D**) Validation of the OPLS‑DA models. R² indicates the explanatory power of the model, and Q² indicates its predictive ability. The X‑axis represents model accuracy, and the Y‑axis represents the frequency of the model classification effect.

(**E**-**F**) Score plots of OPLS‑DA models for YR and GW after *P. litchii* inoculation compared with mock treatment, respectively.

**Supplementary Fig S4. PTOX inhibits the mycelial growth of *P. litchii* on CJA medium.**

Nystatin, a polyene macrolide antifungal antibiotic, was used as the positive control. DMSO served as the negative control.

**Supplementary Fig S5. Plaque areas of *P. litchii* on CJA medium supplemented with different concentrations of PTOX.**

Data are means ± SD of three biological replicates.

**Supplementary Fig S6. Mass spectral analysis and identification of PTOX in YR and GW lychee.**

**Supplementary Fig S7. Determination of PTOX content in naturally grown leaves and axenic seedling leaves following LDB treatment.**

(**A**) Axenic seedlings. (**B**) PTOX content determination.

**Supplementary Fig S8. Venn diagram showing the number and overlap of DEGs.**

DEGs were identified with q-value < 0.001 and fold change > 2.

**Supplementary Fig S9. Functional enrichment analysis of DEGs in YR vs. GW under LDB stimulation.**

(**A**) Gene Ontology (GO) classification of DEGs in biological process (BP) and molecular function (MF) categories.

(**B**) KEGG enrichment analysis of DEGs. The size of the dots corresponds to the number of DEGs enriched in each pathway.

**Supplementary Fig S10. Gene expression analysis of PTOX biosynthesis genes in lychee fruits (A) and leaves (B).**

The data are the mean ± SD of three biological replicates.

**Supplementary Fig S11. Evaluation of LDB resistance in different lychee cultivars.**

Capital letters represent cultivar abbreviations: YR, Yurong; SFZ, Shangfanzhi; GW, Guiwei; HZ, Huaizhi; HML, Honemili; SYH, Sanyuehong; DZ, Dazao; NMC, Nuomici; HDL, Hongdenglong; CZ, Chenzi; SD, Shuidong; ZM, Zaomi.

**Supplementary Fig S12. Alignment of *LcOMT1* promoter sequences.**

**Supplementary Fig S13. Overexpression of *LcOMT1* enhances GW resistance to LDB.**

(**A**) Establishment of an *Agrobacterium rhizogenes*-mediated genetic transformation system for *LcOMT1* overexpression in lychee hairy roots. Red roots indicate transgenic hairy roots expressing the RUBY reporter.

(**B**) Phenotypic analysis showed that *LcOMT1* overexpression significantly enhanced resistance to *P. litchii*

(**C**) qRT-PCR analysis of *LcOMT1* expression levels in transgenic hairy root lines, with GW as the control.

(**D**) Quantification of PTOX content in *LcOMT1*-overexpressing hairy roots.
