# Supplementary material for: Deciphering podophyllotoxin-mediated defense against downy blight in Litchi chinensis Sonn. via integrated metabolomics and transcriptomics
Source: Mol Hortic. 2026 Sep 2;6:63. doi: 10.1186/s43897-026-00257-4 (PMC13536555; doi:10.1186/s43897-026-00257-4)
Supplement: Supplementary file 2 [file 43897_2026_257_MOESM3_ESM.docx]

**Table S2.** Sequence of primers used in this study.

| Primer name | 5’-3’ sequence | Purpose | Vector |
| --- | --- | --- | --- |
| ACTIN_F | TATTGCCGACCGTATGAG | qPCR |  |
| ACTIN_R | AGAGATGCGAGGATTGAT | qPCR |  |
| LITCHI003007_F | ATGCAAAGCTGTGGTCTGCT | qPCR |  |
| LITCHI003007_R | TGCCCTAAAACACGAGGGAA | qPCR |  |
| LITCHI007654_F | TTGGGGCATAACCCGTTACC | qPCR |  |
| LITCHI007654_R | AACACTCCAAGCCCTCATGG | qPCR |  |
| LITCHI010078_F | CACCGATCTCCATCATGCCA | qPCR |  |
| LITCHI010078_R | AACATGAAGCTGCCAAGCAC | qPCR |  |
| LITCHI012082_F | TCATCTCCCCACAAGCAACC | qPCR |  |
| LITCHI012082_R | TGTATGCAGGTGCAAGACCG | qPCR |  |
| LITCHI017676_F | GTGTGTACCCACGAGAACCC | qPCR |  |
| LITCHI017676_R | CAGCATGGTTACGAACTGCC | qPCR |  |
| LITCHI019769_F | ATGCACTTGAACCAGGGAGG | qPCR |  |
| LITCHI019769_R | TGAGGAACTGCGTAGGAGGA | qPCR |  |
| LITCHI019770_F | TCGAGGCCATCAAACTGGTC | qPCR |  |
| LITCHI019770_R | CTGGGTCCATCCCAAACTCC | qPCR |  |
| LITCHI020906_F | TCCGCGATGTTATGAGGCAA | qPCR |  |
| LITCHI020906_R | AAGGTCGCTGTGTCATCCAG | qPCR |  |
| LITCHI021780_F | AGTCCTGGAAGGAGGGGTTC | qPCR |  |
| LITCHI021780_R | ATCTCCAGCATCTTTCGCGT | qPCR |  |
| LITCHI023078_F | GCAGAAGGTGCAAATGGCAA | qPCR |  |
| LITCHI023078_R | AAATTTTGACGGGCGGCTTC | qPCR |  |
| LITCHI024820_F | GACTACCAGGGTCACAAGCC | qPCR |  |
| LITCHI024820_R | ACCAATGCCGGAGCTAACAA | qPCR |  |
| LITCHI026777_F | CCTGCCAATGGATCTGGGAA | qPCR |  |
| LITCHI026777_R | CGCGTGCATTTCATGGATCT | qPCR |  |
| LITCHI027970_F | GAGGAAACATACGGCCCCAA | qPCR |  |
| LITCHI027970_R | AAAGAGCTGGTCGAGTGTGG | qPCR |  |
| LITCHI028719_F | GCTTATGCGGGAAGTGCTTG | qPCR |  |
| LITCHI028719_R | CTTCACCCAAAGGCAACAGC | qPCR |  |
| LITCHI028720_F | TTGTGCACACTTATTGCCGC | qPCR |  |
| LITCHI028720_R | TGAGTGGCTTTCTGGAGTGC | qPCR |  |
| LITCHI031673_F | TCCCAAACTCAGCTCACGTC | qPCR |  |
| LITCHI031673_R | TGCCAATGCTTTTGAACCCG | qPCR |  |
| pOMT1:GUS-F | gtcgacggtatcgataagcttGAAGTATTATGAAGGCAAGTAT | Cloning | pGreen |
| pOMT1:GUS-R | gtatgggtaactagaactagtGTGGACTTGTTGATTATCAGTGT | Cloning | pGreen |
| 35S:OMT1-GFP-F | gggcgcgccctaggaactagtATGAACTCCAAGGAAAGCCAAA | Cloning | pDOE |
| 35S:OMT1-GFP-R | actcatgcgatcgctactagtAGAAACTTTTATAGAGCTCCAC | Cloning | pDOE |
| 35S:OMT1-RUBY-F | gggcgcgccctaggaactagtATGAACTCCAAGGAAAGCCAAA | Cloning | pDOE |
| 35S:OMT1-RUBY-R | ggtcgcatgatccatactagtGAAACTTTTATAGAGCTCCAC | Cloning | pDOE |
| pOMT1:OMT1-GFP-F | atttgaaaaatctcagaattcGAAGTATTATGAAGGCAAGTATATGT | Cloning | pDOE |
| pOMT1:OMT1-GFP-R | actcatgcgatcgctactagtGAAACTTTTATAGAGCTCCAC | Cloning | pDOE |
