# Supplementary material for: Deciphering podophyllotoxin-mediated defense against downy blight in Litchi chinensis Sonn. via integrated metabolomics and transcriptomics
Source: Mol Hortic. 2026 Sep 2;6:63. doi: 10.1186/s43897-026-00257-4 (PMC13536555; doi:10.1186/s43897-026-00257-4)
Supplement: Supplementary file 3 [file 43897_2026_257_MOESM4_ESM.docx]

**Table S3**. Descriptive statistics and ANOVA analysis of DIs in 288 lychee accessions.

| Trait | Year | Mean | SD | Median | Min | Max | G | Y |
| --- | --- | --- | --- | --- | --- | --- | --- | --- |
| Fruit | 2021 | 66.28 | 21.60 | 68.87 | 3.33 | 100 | ** | ** |
|  | 2022 | 54.13 | 23.24 | 55.10 | 0.00 | 100 |  |  |
| Leaf | 2021 | 64.99 | 20.41 | 67.78 | 2.22 | 100 | ** | * |
|  | 2022 | 66.14 | 21.03 | 68.61 | 2.22 | 100 |  |  |
