# Supplementary material for: Deciphering podophyllotoxin-mediated defense against downy blight in Litchi chinensis Sonn. via integrated metabolomics and transcriptomics
Source: Mol Hortic. 2026 Sep 2;6:63. doi: 10.1186/s43897-026-00257-4 (PMC13536555; doi:10.1186/s43897-026-00257-4)
Supplement: Supplementary file 4 [file 43897_2026_257_MOESM6_ESM.docx]

**Table S5.** Quality control of transcriptomes from GW and YR leaves under Mock (sterile water) or LDB treatment.

| Sample | Clean Reads | Clean Bases | Q30 | GC Content |
| --- | --- | --- | --- | --- |
| GW_M1 | 47427854 | 7.11G | 94.56% | 44.57% |
| GW_M2 | 54156422 | 8.12G | 94.59% | 44.69% |
| GW_M3 | 54047850 | 8.11G | 94.70% | 44.69% |
| GW_*P*1 | 55178576 | 8.28G | 94.60% | 44.70% |
| GW_*P*2 | 49424172 | 7.41G | 94.44% | 44.64% |
| GW_*P*3 | 46928690 | 7.04G | 94.12% | 44.66% |
| YR_M1 | 45827120 | 6.87G | 94.06% | 44.42% |
| YR_M2 | 47001924 | 7.05G | 94.17% | 44.37% |
| YR_M3 | 47101146 | 7.07G | 94.25% | 44.44% |
| YR_*P*1 | 45125744 | 6.77G | 94.61% | 44.14% |
| YR_*P*2 | 46380966 | 6.96G | 94.34% | 44.33% |
| YR_*P*3 | 42655620 | 6.4G | 94.57% | 44.52% |

The three biological replicates for each sample were marked with1, 2 and 3 respectively.

Mock, sterile water treatment; *P*, *P. litchii* treatment
